# Hypoxia Pathway Proteins are Master Regulators of Erythropoiesis

**DOI:** 10.3390/ijms21218131

**Published:** 2020-10-30

**Authors:** Deepika Watts, Diana Gaete, Diego Rodriguez, David Hoogewijs, Martina Rauner, Sundary Sormendi, Ben Wielockx

**Affiliations:** 1 Institute of Clinical Chemistry and Laboratory Medicine, Technische Universität Dresden, 01307 Dresden, Germany; deepika.watts@ukdd.de (D.W.); Diana.Gaete@ukdd.de (D.G.); diego.rodriguez@mailbox.tu-dresden.de (D.R.); sundary.sormendi@ukdd.de (S.S.); 2 Section of Medicine, Department of Endocrinology, Metabolism and Cardiovascular System, University of Fribourg, 1700 Fribourg, Switzerland; david.hoogewijs@unifr.ch; 3 Department of Medicine III and Center for Healthy Aging, Technische Universität Dresden, 01307 Dresden, Germany; Martina.Rauner@uniklinikum-dresden.de

**Keywords:** hypoxia, erythropoiesis, EPO, HIF, CKD

## Abstract

Erythropoiesis is a complex process driving the production of red blood cells. During homeostasis, adult erythropoiesis takes place in the bone marrow and is tightly controlled by erythropoietin (EPO), a central hormone mainly produced in renal EPO-producing cells. The expression of EPO is strictly regulated by local changes in oxygen partial pressure (pO_2_) as under-deprived oxygen (hypoxia); the transcription factor hypoxia-inducible factor-2 induces EPO. However, erythropoiesis regulation extends beyond the well-established hypoxia-inducible factor (HIF)–EPO axis and involves processes modulated by other hypoxia pathway proteins (HPPs), including proteins involved in iron metabolism. The importance of a number of these factors is evident as their altered expression has been associated with various anemia-related disorders, including chronic kidney disease. Eventually, our emerging understanding of HPPs and their regulatory feedback will be instrumental in developing specific therapies for anemic patients and beyond.

## 1. Introduction

Red blood cell (RBC) production mainly takes place in the bone marrow during homeostasis. This process encompasses a number of proliferation and differentiation steps, starting at the hematopoietic stem cell (HSC) and down to specialized erythroid progenitors, finally turning into mature RBCs. The complex process of erythropoiesis is vastly intertwined with iron metabolism [1] and regulated by a number of cytokines, including the central glycoprotein hormone erythropoietin (EPO). The primary producers of EPO are specialized fibroblast-like peritubular cells located in the adult kidney or fetal hepatocytes. Nevertheless, more recent studies have also reported EPO production in astrocytes, osteoblasts, and pericytes upon transcriptional response, dependent on the hypoxia pathway [2,3,4,5,6]. Our initial understanding of this pathway came into light almost 30 years ago when the group of Dr. Semenza identified hypoxia-inducible factor (HIF) as a transcription factor responsible for the expression of EPO [7,8]. Hence, increased EPO during diminished oxygen supply, for instance, at high altitude, causes our body to make more RBCs to augment the delivery of oxygen. Since then, numerous hypoxia pathway proteins (HPPs) have been identified in different cells of our body, often playing critical roles in survival, growth, and differentiation upon changes in pO_2_ [9]. Cellular oxygen sensing is, therefore, a fundamental biological process that is necessary for the adaptation of living organisms to changing physiological and pathological situations. To date, a limited amount of enzymes, three HIF prolyl hydroxylase domain proteins (PHD1–3), and factor-inhibiting HIF (FIH) have been identified as oxygen sensors. These oxygen-dependent enzymes act as hydroxylases and dioxygenases, regulating the availability of HIFα subunits, and, in turn, define when and how cells express genes in response to changing pO_2_ [10,11,12]. Under hypoxia, when the oxygen sensors are inactive, HIFα subunits are stabilized and translocate to the nucleus and bind to HIFβ and a number of other cofactors. Subsequently, the HIF-complex binds hypoxia response elements (HREs) in the promoter/enhancer of a selection of genes, generally increasing their transcription [13,14,15]. This review will give a more indepth view on the role of HPPs (i.e., HIFα, PHDs, HIF-controlled genes) regulating the multistage process of erythropoiesis and discusses the current therapeutic approaches interfering with the hypoxia pathway in the treatment of anemia and/or chronic kidney disease (CKD) [16].

## 2. HIF Transcription Factors and Their Modulators

The heterodimeric HIF-complex is a key transcription factor constituted of an α subunit and a β subunit. The latter, also referred to as aryl hydrocarbon receptor nuclear translocator (ARNT), is constitutively expressed, whereas HIFα (i.e., HIF1α, HIF2α (also known as endothelial PAS domain containing protein 1 (EPAS1), and HIF3α) is synthesized and constantly degraded during normoxia. Degradation of the HIFα subunits is tightly regulated by three PHDs (also known as EGL-9 homolog EGLN2, EGLN1, and EGLN3, respectively) that hydroxylate two specific proline residues (Pro402/Pro564 in HIF1α and Pro405/Pro531 in HIF2α) in the oxygen-dependent degradation domain [11,17,18,19]. Consequently, this hydroxylation leads to von Hippel–Lindau tumor suppressor protein (VHL) mediated ubiquitination and proteasomal degradation of the HIFα subunits [12,19,20].

Although a recent in vitro study demonstrated that HIFs are the only targets of the PHDs [21], each PHD has a different affinity for a particular HIF subunit: HIF2α is mainly regulated by PHD1, PHD2 is the major regulator of HIF1α [22,23]. Noteworthy, PHD2 is the most widely expressed PHD isoform, being of crucial importance during development. Indeed, early studies in mice reported that PHD2 deficiency results in embryonic lethality between E12.5 and E14.5 due to defects in the developing placenta and heart [24,25]. Conversely, PHD1 and PHD3 deletions are not lethal and only have a limited tissue-specific impact, as exhibited by their effect on cellular metabolism in skeletal muscle and altered blood pressure in the central nervous system, respectively [26,27]. Moreover, PHD2 differs from PHD1 and PHD3 in its N-terminal zinc finger, which favors the recruitment of HSP90 machinery, facilitating HIFα hydroxylation [28,29]. In turn, PHD1 and PHD3 can also be regulated by hypoxia-inducible E3 ubiquitin ligases—the SIAH proteins [30]. HIFα’s availability can be further modulated at the transcriptional level through crosstalk with other signaling pathways and epigenetic regulators. For instance, nuclear factor­κB (NF­κB) and signal transducer and activator of transcription 3 (STAT3) have been shown to bind the HIF1α promoter and induce its transcription [31,32]

Whereas HIF1α is expressed ubiquitously, HIF2α is more restricted to specific cell types like endothelial cells, cardiomyocytes, hepatocytes, adipocytes, neurons, and interstitial cells and glomeruli of the kidney [21,33,34,35,36,37]. Even though both HIFs regulate many common genes, their transcriptional targets often differ, as well as their kinetics of activation and oxygen dependence. Overall, gene expression and chromatin immunoprecipitation assays showed that both HIF1α and HIF2α are responsible for regulating the transcription of more than a thousand genes during the adaptive response to hypoxia [38,39,40].

Conversely, hypoxia has also been reported to suppress the expression of a number of genes by activation of transcriptional repressors or epigenetic regulators. The hypoxia-mediated gene suppression can be either HIF-dependent or HIF-independent. For instance, Cavadas and colleagues recently showed that hypoxia-mediated HIF-independent nuclear translocation of repressor element 1-silencing transcription factor (REST) is responsible for regulating 20% of hypoxia-repressed genes in hypoxia [41]. The other transcriptional repressors induced by hypoxia in a HIF-dependent manner include DEC1, DEC2, ID2, Zeb1, and Zeb2, as reviewed by Cavadas et al. [42]. Hypoxia also regulates epigenetic factors, such as DNA methylation and histone acetylation; those, in turn, regulate the hypoxia-responsive gene expression [43]. In addition, several recent studies, including ours, have pointed towards HIF-mediated suppression by microRNAs [44,45,46,47,48]. Interestingly, there is two-way communication between HIFs and microRNAs, as the latter have also been suggested to control the expression of HIFs in different cell types [49,50] (recently reviewed by Serocki and colleagues [46]).

Soon after their discovery, PHDs have been considered to play a distinctive role in regulating erythropoiesis. Somatic inactivation of PHD2 after birth causes a significant rise of serum EPO levels, an effect that can only be mimicked by the simultaneous deletion of PHD1 and PHD3. Importantly, we and others discovered that this phenotype is exclusively dependent on HIF2α since its targeted deficiency, but not that of HIF1α, reverses this phenotype [51,52]. Briefly, severe HIF2-driven EPO production resulted in nonlethal hematocrit levels up to 85% upon PHD2 deletion in CD68-expressing cells, including renal EPO-producing cells (REPCs) [34]. In humans, various heterozygous point mutations have been described in the PHD2 gene, resulting in an increase of red blood cell mass, hemorrhages, strokes, and an enhanced risk of thrombosis [53,54,55]. The mutations P317R and P371H affect the substrate binding of PHD2, partially inhibiting the hydroxylation of HIF [56]. Interestingly, a patient with a heterozygous H374R mutation developed a recurrent paraganglioma that exhibited loss of PHD2 in both alleles in addition to the erythrocytosis [53].

**Figure 1:**
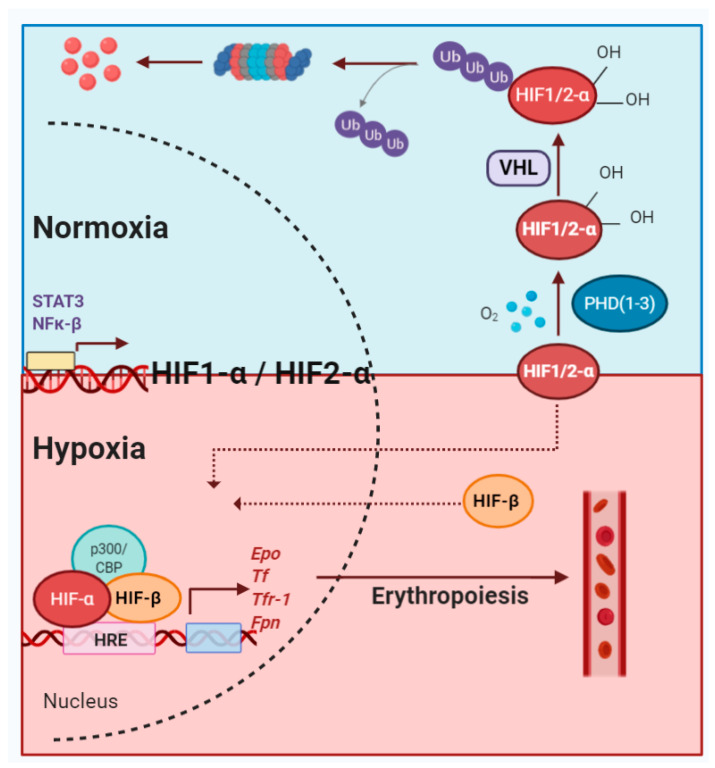
Schematic overview of the hypoxia pathway under normoxia and hypoxia. The hypoxia-inducible factor (HIF)-α subunits are synthesized and subsequently degraded under normoxia (blue). In the presence of physiological oxygen levels, prolyl hydroxylase domain proteins (PHDs) mediate hydroxylation of HIFs, leading to subsequent binding to von Hippel–Lindau tumor suppressor protein (VHL). VHL facilitates the ubiquitination of hydroxylated HIFs, resulting in proteasomal degradation. However, under hypoxia (red), the hydroxylation of the alpha subunits is inhibited, stabilizing the HIF-α subunits. The stabilized alpha subunits bind to the beta subunit and translocate to the nucleus. The HIFs, together with other cofactors, bind hypoxia response elements (HREs), promoting the transcription of genes essentially involved in erythropoiesis. Created with Biorender.com.

Next to EPO and its receptor, several other genes involved in the process of erythropoiesis are directly or indirectly regulated by the PHD/HIF axis. These include genes implicated in iron metabolism, such as transferrin (*Tf*), transferrin receptor-1 (*Tfr-1*), ferroportin (*Fpn*), hepcidin, divalent metal transporter 1 (DMT1), duodenal cytochrome b (DCYTB), and GATA-1 [56,57,58,59,60,61], granting this axis a central role during anemic processes associated to a wide range of diseases (Figure 1). The regulation of various genes involved in the erythropoiesis regulated by hypoxia or HPPs is summarized in Table 1.

## 3. Erythropoiesis: A Multistep Process

On average, 200 × 10^9^ RBCs are generated and released into circulation every day [64]. This production is adjusted on a need basis and is tightly controlled by complex networks involving HPPs, regulators of iron metabolism, and erythropoietic stress [1,84,85,86]. Erythropoiesis is a multistep process that includes numerous proliferation and differentiation stages, starting from the HSC and down to committed erythroid progenitors and different erythroblast populations in the bone marrow (BM). This eventually gives rise to reticulocytes and enucleated mature RBCs in the blood [7,64,87]. The course of this development includes strict control by a number of transcription factors and epigenetic regulators. GATA-1 is the major transcription factor regulating the differentiation and survival of erythroid progenitors [63,88,89,90], including genes involved in heme and/or globin synthesis, antiapoptosis, cell cycle regulators, and the erythropoietin receptor (EPOR) [88,89,90,91,92,93,94]. In turn, GATA-1 is also regulated by HIF [62,93,95]. Additionally, GATA-2 is essential for the regulation of lineage-restricted gene expression during erythroid differentiation [63,96]. Other erythropoietic regulators that have been studied in the context of hypoxia and HPPs are interleukin-3, certain vitamins, and transferrin (TF)-transferrin receptors (TFR) modulating iron metabolism [59,60,61].

Several studies in the field of erythropoiesis have also explored the role of micro-RNAs, including during the process of enucleation [65,97,98,99]. A recent study by Rivkin and colleagues reported that the hematopoietic-specific mir-142 plays a critical role in maintaining the typical biconcave shape of RBCs through the control of cytoskeletal regulators and directly influencing the overall lifespan of the erythrocyte [100]. In another study, mir-142 was shown to target HIF1α directly [101]. Although more research is required to unambiguously link mir-142, HIF1α, and the shape of the RBCs, other studies have reported HIF’s participation in the regulation of micro-RNAs involved in different stages of erythropoiesis [65,98,102,103] (Table 1).

## 4. EPO/EPOR Axis: Hypoxia Pathway in Action

EPO is the central hormone during erythropoiesis and is mainly responsible for survival, proliferation, and differentiation of committed progenitors, ultimately enhancing the oxygen-carrying capacity of blood [104]. EPO promotes the survival of colony-forming unit-erythroid cells (CFU-E) and early erythroblasts. Moreover, erythroid precursors are differentially sensitive to EPO and respond differently to increasing levels. At higher levels, cells survive and differentiate, whereas at lower EPO levels, less sensitive cells die due to caspase-mediated apoptotic death [91].

After birth, EPO is mainly produced by REPCs, whereas hepatocytes are the main source during embryo development [105,106,107,108,109,110,111]. In the BM, EPO binds to its homodimeric receptor EPOR on erythroid progenitor cells, triggering its association with Janus kinase (JAK2). In turn, JAK2 phosphorylates the cytoplasmic tail of EPOR, providing multiple docking sites for signal-transducing proteins, which subsequently lead to STAT-5 phosphorylation [106]. Additionally, it was shown that the phosphatidylinositol-3-kinase/protein kinase B (PI-3k/AKT) and mitogen-associated protein kinase/extracellular signal-related kinase (MAPK/ERK) pathways can also be activated in this way [105].

Severe hypoxia or loss of PHD2 in hepatocytes can also result in dramatic liver EPO production in adults [68,111,112,113]. However, in contrast to HIF2α-mediated EPO production, HIF stabilization in the epithelial cells of the kidney results in suppression of EPO, leading to anemia. The lab of Dr. Haase showed that proximal nephron-specific deletion of VHL (Pax8-rtTA–cre) results in a reduction of REPCs and renal anemia, suggesting cellular crosstalk in the tubule–interstitial compartment in the kidney, changing the production of EPO [114].

Since HIF2α is a major player in erythropoiesis, most of the studies have focused on this subunit as a facilitator of EPO production. Interestingly, HIF3α was also recently shown to be involved in EPO regulation [115,116,117]. Until now, only limited studies have provided insight into the complex alternative splicing of *HIF3α* mRNA, resulting in short and long variants either inhibiting the hypoxia response or possessing transactivation capacities [116,118]. In vitro knockdown or overexpression of the long HIF-3α-2 splice variant in EPO-producing cell lines resulted in the downregulation or upregulation of EPO, respectively, involving the canonical HRE [115,116]. However, further research is required to define whether HIF3-mediated EPO regulation also occurs in vivo.

Whereas HIF2 dependent EPO regulation is well-established, the precise molecular mechanism responsible for tight expression control and the regions within the EPO locus that are responsive to HIF-mediated regulation remain the subject of continuous research efforts. It is known that oxygen-dependent regulation of the EPO gene is controlled by distinct regulatory sequences in the liver and kidney and involves a region of 0.4 kb of the 5′-sequence (promoter) and a 0.7 kb region of the 3′-sequence in the liver [9,119] whereas a highly conserved regulatory element responsible for renal EPO expression has been suggested to be located between 14 and 6 kb in the 5′-region [120,121]. However, the strict tissue-specific and conditional EPO transcriptional regulation strongly suggests that additional mechanisms might be involved. Moreover, studies on a single gene scale [122] and at the pangenomic level [123] showed that long-range enhancer-promoter chromatin looping occurs independently of HIF and involves the recruitment of transcriptional factors and coactivators, further suggesting that EPO regulation might be more complex. Accordingly, Smythies et al. [40] illustrated that the tissue-specific role of HIF2α is related to association with tissue-specific transcription factors. More recently, Orlando et al. reported that multiple distal and proximal HREs cooperate in oxygen-regulated *EPO* gene expression and that EPO regulation in renal cells may have more in common with neuronal cells than with hepatic cells, further illustrating the context-dependent complexity of EPO regulation [124].

## 5. Hypoxia-Mediated EPO Production at Other Sites

Apart from REPCs and embryonic hepatocytes, other cell types have also been shown to produce EPO in response to hypoxia. As an example, hypoxia-induced EPO production in the brain and certain cell types of the central nervous system (CNS) have come to light in the past years [125,126]. Urrutia and colleagues showed that pericyte specific (NG2:cre) VHL or PHD2/PHD3 deficiency leads to polycythemia. This was reversed in mice that were additionally lacking HIF2α, emphasizing the essential role of HIF2α for EPO production in other cell types [5,125,127]. Rankin and colleagues showed that specific deletion of VHL in osteoprogenitors in mice (OSX:cre-VHLf/f) presented elevated EPO expression in bone, which was also accompanied by polycythemia. Additionally, in this setting, HIF2α was the central HIF isoform [4].

It is noteworthy that the majority of EPO-producing cells, such as pericytes, astrocytes, as well as REPCs, are of neurological origin [5,6,52,128]. Recent unpublished data from our lab also described EPO production in the adrenal gland chromaffin cells upon HIF2α activation, cells known to be derived from the neural crest. In line with this, polycythemia is one of the initial traits recognized in pheochromocytoma (the tumor of the adrenal medulla) relate to VHL or loss-of-function mutations or HIF2 gain-of-function mutations [67]. In addition to HIF-mediated increased EPO expression in tumors, EPOR is also significantly increased in the hypoxic region of tumors, suggesting a HIF-mediated modulation of EPOR [69,129].

## 6. EPOR and HIF Axis

EPO will bind to erythroid progenitors (i.e., erythroblasts) in the BM/spleen via the EPOR to meet the enormous daily production of RBCs. Remarkably, an in silico study identified almost thirty genetic variants of the EPOR and seven variants of the EPO gene associated with erythrocytosis or increased hematocrit in humans. Mutations in EPOR have been shown to activate the receptor constitutively, resulting in enhanced production of erythroid colonies and erythrocytosis but reduced EPO synthesis [130].

In addition to its expression on erythroblasts, EPOR has even been identified on nonerythroid cells such as neural cells, endothelial cells, skeletal muscle myoblasts, macrophages, and white adipose tissue [131,132,133]. Although under normal conditions, EPOR expression is very low in the nervous system, mild episodes of hypoxia can significantly increase its expression [70]. Similarly, unpublished data from our lab suggest HIF-mediated EPOR expression in the adrenal medulla.

Furthermore, Su and colleagues recently showed that hypoxia induces EPOR expression via HIF1α in lung carcinoma cells. According to this study, HIF1α interacts with the transcription factor early growth response 1 (EGR1) to negatively regulate EPOR in the early phase of hypoxia, and with SP1 to promote EPOR expression in the later phase [69]. Thus, the hypoxia pathway is crucial in modulating the expression of EPO and its receptor. Both are not only directly involved in erythropoiesis; EPO is also crucial in the regulation of the iron metabolism via hepcidin suppression [80] as discussed further in the review.

## 7. Iron Metabolism: HPP as Cucial Contributors

**Figure 2:**
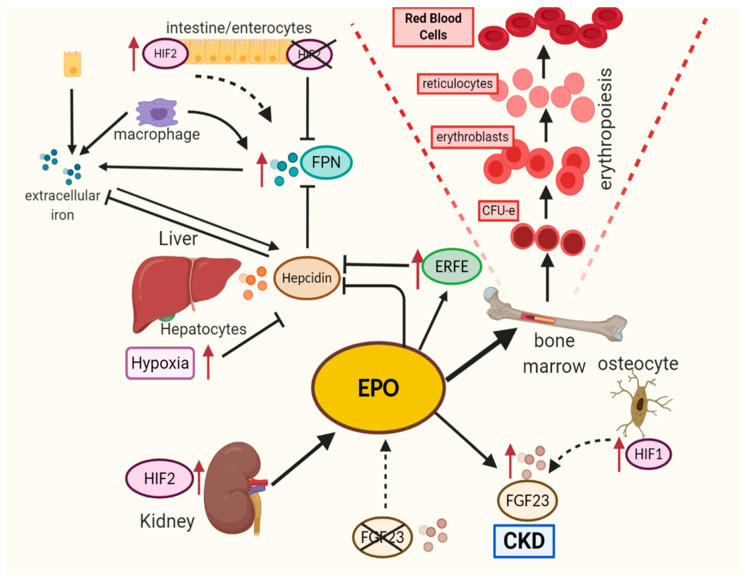
Hypoxia pathway proteins mediated regulation of erythropoiesis. EPO, the crucial factor involved in erythropoiesis, is mainly HIF2-dependently regulated in the kidney. EPO produced in the kidney acts on erythroblasts in BM via EPOR and is essential in the development of red blood cells, especially in the late phases. In addition to its crucial role in the development of RBCs, iron metabolism is indispensable. More information can be found in the text. Created with Biorender.com.

Hemoglobin is the most essential component of erythrocytes as it carries oxygen bound to heme groups throughout the body. Central in heme is iron, which makes up about 70% of all available iron. Therefore, iron transport and metabolism are of utmost importance and, in turn, are also regulated by a number of HRE-containing genes, directly linking it to the hypoxia pathway [134]. For instance, to maintain necessary iron levels in other cells, HIF2α plays a pivotal role in promoting cellular iron uptake. Particularly, HIF2α upregulates divalent metal transporter 1 (DMT1) and duodenal cytochrome b (DCYTB), where DMT1 is responsible for iron transport into cells, while DCYTB reduces the ferric form of iron to ferrous iron [135,136]. HIFs also regulate the expression of transferrin (TF) and transferrin receptor-1 (TFR-1), where TF catches up to two iron atoms from circulation and transports it into the cells via TFR-1 [60,61,137,138]. FPN, a critical factor that facilitates iron export from iron-storing cells (i.e., enterocytes and macrophages), is inhibited by the peptide hormone hepcidin [139]. Therefore, it is crucial that hepcidin production is strictly controlled for normal erythropoiesis to occur. To ensure this, erythroblasts produce and secrete the hormone erythroferrone (ERFE) upon EPO signaling, suppressing hepcidin production [77,79,80,138,140,141]. A more recent study by Arezes and colleagues suggests direct interaction of ERFE with BMP5, BMP6, and BMP7, leading to the inhibition of BMP/SMAD signaling and consequent hepcidin suppression [79] (Figure 2 and Table 1). More detailed insights into the regulation and action of ERFE have been recently reviewed by Coffey and Ganz [140].

Interestingly, hepcidin functionality has been linked to HIF2α activity [75,78]. Initial studies have pointed towards a direct role of HPP during hepcidin production, where hypoxia inversely regulates hepcidin expression [142] by downregulating the cyclic AMP response element-binding protein H (CREB-H), mediated by platelet-derived growth factor (PDGF-BB) [74] or direct suppression of hepcidin by HIF binding to HRE, and suppressing *HAMP* (encoding hepcidin) transcription [143]. Vice versa, the deletion of hepatic hepcidin significantly increased FPN, leading to cellular iron efflux, decreased PHD activity, and, eventually, HIF2α upregulation [75].

Further, a negative feedback loop of EPO by increased iron levels has been proposed. Iron excess, induced by injection or feeding, resulted in decreased renal EPO gene expression, which is mediated through reduced HIF2α levels in renal interstitial fibroblasts [144]. In addition to regulation by PHDs, translational regulation of HIF2α is inhibited by binding of iron regulatory proteins (IRPs) on iron response element (IRE) in its 5′UTR [113,145]. Taken together, these studies suggest a Fe–HIF2α–EPO–ERFE–hepcidin axis, underlining the importance of HIF2α in iron metabolism [75,146,147]. PHDs and HIFs can be potentially targeted for the treatment of iron-related disorders such as anemia, chronic kidney disease (CKD), and polycythemia, whereas HIF2α is an essential target for polycythemia-related disorders [106].

Chronic kidney disease (CKD) is one of the leading causes of death and arises mainly as a result of diabetes and hypertension [148]. Although anemia can be caused by a large variety of reasons, anemia due to kidney damage is attributed to decreased production of EPO by the kidneys, leading to suppression of erythropoiesis [107]. HIF stabilizers act by restoring EPO levels and reducing hepcidin and have been used effectively to enhance hemoglobin levels in patients suffering from anemias, including CKD patients [149].

## 8. Anemia and CKD—The PHD–HIF Axis and Therapeutic Targeting

Anemia can have different etiologies and is a common feature during the course of numerous pathologies. As such, the success of specific anemia treatments depends on the disease context. In many cases, including CKD-related anemia, treatment includes erythropoiesis-stimulating agents (ESA) [91]. However, there is not always a clear inverse correlation between the degree of anemia and EPO levels in the serum. In fact, many patients suffering from renal anemia do not respond to treatment with ESA [82]. Together, this suggests that modulating EPO levels in serum is not sufficient to treat anemia in CKD patients and that new therapeutic approaches need to be developed. In line with this, a recent study showed that anemia in rats led to increased expression of HIF2α and EPO in kidney and bone marrow, whereas rats suffering from CKD failed to show these increases.

An important factor contributing to the progression of CKD is incomplete or maladaptive tissue repair following acute kidney injury (AKI). In this setting, HIF stabilization through pharmacological PHD inhibitors enhances kidney recovery in several types of AKI, including AKI caused by nephrotoxicity and renal reperfusion, the most common causes of AKI. Importantly, other studies suggest that HIF1α accumulation might be harmful during sepsis-associated AKI. This indicates that the success of HIF stabilization depends on the type of AKI and, possibly, also other factors [150].

Since PHDs need 2-oxoglutarate (2-OG) for their hydroxylase activity, most of the clinically advanced HIF stabilizers are 2-OG derivatives (reviewed by [151]). To date, several drugs that inhibit PHD activity have progressed into clinical trials. Importantly, these drugs do not target FIH, which preferentially inhibits HIF1α transcriptional activation, allowing for increased HIF2α stabilization. HIF stabilization induces a transient increase in EPO levels, achieving concentrations closer to the physiological range than those obtained with current ESA therapy. This suggests that treatment with HIF stabilizers could be more suited for certain patients (for recent reviews, see [152,153,154,155]. Despite the promising future HIF stabilizers hold for the treatment of anemia in CKD patients, concerns have arisen regarding their chronic use because of the significant pool of genes targeted by HIFs as well as the higher risk of hyperkalemia or the development of iron deficiency [152,156,157]. Therefore, further research is of utmost importance to better understand the impact of long-term HIF stabilization and to identify drugs targeting HIF1α and/or HIF2α isoforms specifically.

## 9. HIF–EPO–FGF-23 Axis: PHD2/HIF Inhibition

As discussed above, HIF-dependent EPO regulation is crucial for appropriate erythropoiesis in homeostatic and pathological conditions. Considering that adult erythropoiesis occurs in BM, it is noteworthy to mention another important player in this axis: fibroblast growth factor-23 (FGF-23). FGF-23 is a bone-derived hormone essential for regulating vitamin D and phosphate concentrations [158]. Two forms of FGF-23 have been described: a full-length biologically active protein (iFGF-23) that, upon cleavage, results in a C-terminal inactive fragment (cFGF-23) [159,160]. While iFGF-23 interacts with FGF receptor 1 (FGFR1) to decrease blood phosphate levels in mice, cFGF-23 can directly increase BM erythroid cell numbers in the same manner as treatment with recombinant human (rhEPO) [82]. Accordingly, FGF-23 constitutes the link between erythropoiesis and bone homeostasis in both physiological as well as in a variety of pathological conditions [161,162]. Indeed, we recently showed that increased FGF-23 levels are associated with ineffective erythropoiesis and impaired bone mineralization in myelodysplastic syndromes (MDS) [159]. Similarly, in CKD mouse models, the expression of FGF-23 mRNA is significantly increased in bone cells, resulting in reduced bone mineralization [163]. Additionally, it has been reported that FGF-23 expression can be regulated by inflammation and iron deficiency. Induction of inflammation in mice using IL-1β, LPS, and/or TNF correlates with increased expression levels of FGF-23 in osteocytes, both in humans and in mice [164], but also in CKD and inflammation in other organs [165]. David et al. showed that IL1β-induced inflammation resulted in an FGF-23 increase, accompanied by a significant ferritin increase and a decrease of iron in the serum. Interestingly, the same study demonstrated that increased skeletal FGF-23 expression during inflammation remained even after the administration of the PHD inhibitor FG-4592 [166] (roxadustat—one of the PHD inhibitors currently in clinical trials for CKD treatment [167]). Conversely, another in vitro study in bone derived cell line showed increased FGF-23 mRNA expression upon HIF1α activation [168]. Noonan et al. studied the role of the HIF/EPO/FGF-23 axis in iron metabolism in an early-stage CKD mouse model. They reported that, upon treatment with HIF–PHD inhibitor FG-4592, iFGF-23 serum levels dropped remarkably. As a result, the deficiency of iron metabolism stabilized through decreased liver *ferritin*, *Bmp6*, and *hepcidin* mRNAs [169]. Taken together, FGF-23 constitutes an important regulator of erythropoiesis and bone homeostasis. Although there is supporting evidence linking the regulatory role of HIFs and FGF-23 expression, additional studies are required to unravel the molecular mechanisms linked to these HPPs (Figure 2).

## 10. Conclusions

Taken together, hypoxia pathway proteins play direct and indirect roles during the complex process of erythropoiesis. Therefore, the multifaceted regulation of all these proteins is essential in order to preserve the enormous demand for red blood cells needed under steady-state but also stress situations. The most crucial member of this system is EPO, primarily produced in the kidney, and directly regulated by the PHD2/HIF2α axis. Besides its impact on the development of red blood cells, EPO plays an essential role in iron metabolism by regulating hepcidin expression in the liver. In addition, EPO steers the production and processing of other growth factors (e.g., FGF-23) that are essential during erythropoiesis and, in that way, regulates its own production. Due to the essential roles of HPPs in erythropoiesis, the development of HIF stabilizers and PHD inhibitors as therapeutic agents are under detailed investigation for the treatment of anemias of various etiologies. However, more research is necessary to better understand the wide impact of the central HPPs, especially for the chronic intervention of this pathway.

## Figures and Tables

**Table 1:** Regulators of erythropoiesis.

Stage of Erythropoiesis/Iron Metabolism	Factors	Key Role in Erythropoiesis	Regulation by HIF/EPO	References
Early stages of development of erythroid progenitors	GATA-1	Initiates erythropoiesis Regulates the transcription of several erythroid differentiation-related genes	HRE present in GATA-1 and GATA-1 regulation by HIF1. EPO protects GATA-1 from caspase-induced degradation	[62,63,64,65]
Pro-erythroblasts to late erythroblasts	EPO	Key cytokine essential for growth, survival, and differentiation of RBC’s	HIF2 directly regulates EPO	[52,66,67,68]
	EPOR	Essential for erythropoiesis and action of EPO	Some evidence on regulation by hypoxia and HIFs	[69,70,71]
Late stage maturation/Apoptosis	FAS, FAS-L	Apoptosis and arrest maturation	Downregulation by EPO	[72]
Iron metabolism	Hepcidin	A crucial regulator of iron metabolism; suppresses ferroportin	Direct and indirect suppression by hypoxia, HIF, and EPO	[73,74,75,76,77]
	Ferroportin (FPN)	A critical factor that facilitates iron export from the cells	Regulated by HIF2a and hepcidin	[78]
	Erythroferrone (ERFE)	Suppresses hepcidin production	Upregulation by EPO	[79,80]
	Transferrin (TF)	Required for transporting iron	Regulation by HIF1	[61]
	Transferrin receptor (TFR1)	Plays role in erythroid differentiation/ role in iron uptake	Induced by HIF1 and hypoxia	[59,60]
	FGF-23	Potential role in erythropoiesis. blockage of FGF-23 results in increased erythropoiesis	Increased by EPO	[66,81,82,83]
